# Antibiotic Resistant Bacterial Isolates from Captive Green Turtles and* In Vitro* Sensitivity to Bacteriophages

**DOI:** 10.1155/2017/5798161

**Published:** 2017-09-24

**Authors:** Alessandro Delli Paoli Carini, Ellen Ariel, Jacqueline Picard, Lisa Elliott

**Affiliations:** ^1^College of Public Health, Medical and Veterinary Sciences, James Cook University, 1 Solander Drive, Townsville, QLD 4811, Australia; ^2^Ausphage, Townsville, QLD 4811, Australia

## Abstract

This study aimed to test multidrug resistant isolates from hospitalised green turtles* (Chelonia mydas)* and their environment in North Queensland, Australia, for* in vitro* susceptibility to bacteriophages. Seventy-one Gram-negative bacteria were isolated from green turtle eye swabs and water samples. Broth microdilution tests were used to determine antibiotic susceptibility. All isolates were resistant to at least two antibiotics, with 24% being resistant to seven of the eight antibiotics. Highest resistance rates were detected to enrofloxacin (77%) and ampicillin (69.2%). More than 50% resistance was also found to amoxicillin/clavulanic acid (62.5%), ceftiofur (53.8%), and erythromycin (53.3%). All the enriched phage filtrate mixtures resulted in the lysis of one or more of the multidrug resistant bacteria, including* Vibrio harveyi *and* V. parahaemolyticus*. These results indicate that antibiotic resistance is common in Gram-negative bacteria isolated from hospitalised sea turtles and their marine environment in North Queensland, supporting global concern over the rapid evolution of multidrug resistant genes in the environment. Using virulent bacteriophages as antibiotic alternatives would not only be beneficial to turtle health but also prevent further addition of multidrug resistant genes to coastal waters.

## 1. Introduction

The increasing quantities of antibiotics released in the environment due to anthropogenic activities are selecting for resistant bacterial strains in all environments. Large-scale applications of antibiotics, other than for human therapy, include use in aquaculture and agriculture, use as animal growth promoters, and use for culture sterility in research and industry, as well as other therapeutic and prophylactic use in animal hospitals and rehabilitation centres [1, 2].

Due to the severe debilitation of sea turtles on entry to rehabilitation centres, they are often treated with a broad-spectrum antibiotic both prophylactically and therapeutically against microbial diseases without prior antibiotic susceptibility testing [3]. Broad-spectrum antibiotics can further jeopardise the health of a green turtle* (Chelonia mydas)* by killing the intestinal bacterial flora they rely on for hind-gut fermentation. A damaged intestinal bacterial flora increases the risk of intestinal disease and malnutrition, enhancing the green turtle's susceptibility to bacterial infection and thus eliciting a treatment cascade. Furthermore, the discharge of those antibiotics into aquatic environments via waste waters may destroy or inhibit important environmental bacteria [4, 5].

Recently, bacteriophages or phages have gained increased attention as an alternative to antibiotics and other antibacterial chemicals in order to reduce the spread of multidrug resistant bacteria and control bacterial diseases where antibiotics are no longer effective [6]. Sea turtles' eyes are in direct contact with the surrounding environment and exposed to physical damage and bacterial infections even during captive care [7, 8]. In this study, we isolated and performed antibiotic sensitivity tests on bacterial isolates from hospitalised green turtle eyes. Multidrug resistant isolates were subsequently tested for* in vitro* susceptibility to bacteriophages in order to assess their potential use in rehabilitation settings.

## 2. Materials and Methods

### 2.1. Sampling

A total of seven captive green sea turtles, two of them from the ReefHQ Turtle Hospital in Townsville and five from the Fitzroy Island Rehabilitation Centre in Cairns, were sampled between June and July 2014 in accordance with the following permits: WISP14626814 from the Department of Environmental and Heritage Protection, A2026 Animal Ethics, and G14/36896.1 from the Great Barrier Reef Marine Park Authority. A total of 14 swabs were taken from under the dorsal eyelid of both eyes on each turtle. Water samples were collected from the turtle holding tanks at ReefHQ and Fitzroy Island Rehabilitation Centre. A water sample was collected from the display tank at ReefHQ as hospitalised turtles are often held there. Two one-litre surface water samples were also collected from the coastal environment off Magnetic and Orpheus Islands, North Queensland.

### 2.2. Isolation of Bacteria

The swabs were plated onto five agars, from less selective to most selective, 5% sheep blood (Acumedia, CellBioSciences), marine salts enriched tryptose (Oxoid™), McConkey agar with marine salts (Oxoid), thiosulfate-citrate-bile salts-sucrose agar (TCBS) (Acumedia, CellBioSciences), and marine salts agar with phenylethyl alcohol (Oxoid), and incubated at 30°C overnight. Water samples were filtered through 0.45 *μ*m mesh and 90 mm diameter glass microfibre filters (Whatman™) with a dry vacuum pump/compressor (Model 2511 Welch™), and the filters were cultured for bacteria using the same media and incubation conditions as the eye swabs. Isolated bacterial colonies were selected based on their different morphologies and purified by individually plating each selected colony onto blood agar with marine salts and incubated at 30°C overnight. Purified colonies were grouped using Gram staining, oxidase, catalase, and spot indole tests. Each bacterial isolate was subjected to antibiotic susceptibility tests using broth microdilution plates to detect their susceptibility to antibiotics. Bacterial isolates that showed multidrug resistance (to at least 3 antibiotics) were therefore selected for more specific identification using the following tests: growth on TCBS agar for sucrose fermentation, growth on McConkey agar for lactose fermentation, and motility using the “hanging drop” microscopic technique. Final identification to species level was carried out using Micro Sys™ plates for* Vibrio* spp. or Biolog™ (CellBioSciences Pty Ltd, Heidelberg, VIC) tests.

### 2.3. Broth Microdilution Plate Preparation

Broth microdilution plates were prepared by inoculating 100 *μ*l of artificial sea water (ASW) in each well of a 96-well flat-bottom plate (Corning™). Each well in the first column (A–H) was then inoculated with 100 *μ*l of 8 different analytically pure antibiotics (Sigma-Aldrich™) resulting in initial concentrations shown in Table 1. Twelve 2-fold dilutions of the initial antibiotic concentrations were carried across each row. Broth microdilution plates were stored in a −20°C freezer until required.

### 2.4. Determination of Minimum Inhibitory Concentrations and Epidemiological Cut-Offs

Bacterial isolates were inoculated into sterile Mueller-Hinton broth containing 1.5% salts (from ASW). This culture was incubated in an orbital shaker for 8–10 hours at a temperature of 30°C and a rotation speed of 150 rev min^−1^. Bacterial density was brought to 0.5 McFarland standard by using a spectrophotometer set at 600 nm wavelength. A 100 *μ*l aliquot from the 0.5 McFarland bacterial suspension was then inoculated in each well with an 8-tip multichannel pipette starting from the highest dilution. Inoculated broth microdilution plates were incubated at 30°C overnight. A control plate was inoculated with an ATCC 25922* Escherichia coli* strain in order to assess whether antibiotics performed as expected and check that the marine salts media would not negatively impact on antibiotic performance. Minimum inhibitory concentrations (MICs) were determined using the Clinical and Laboratory Standards Institute (CLSI) method [9]. Epidemiological cut-offs (ECOFFs) were determined as the upper limits of the MICs distribution curves (upper limit of the wild-type bacterial strain). Repeats were subsequently performed with the bacterial isolates that showed resistance to at least three antibiotics.

### 2.5. Bacteriophage Enrichment

Selected bacterial isolates were subcultured in nutrient broth and incubated at 30°C for 5–8 hours in an orbital shaker at a rotation speed of 150 rev min^−1^. Water from the waste water of an extensive prawn farm in Thailand and from a turtle holding tank at ReefHQ was used as water sources for bacteriophage amplification. A 2 ml aliquot of the bacterial broths was combined with 50 ml of nutrient broth (with doubled nutrient and salts concentrations) and 50 ml of water. The protocol was repeated for all the bacterial samples and both the water sources (Thailand and ReefHQ). Cultures were then incubated in an orbital shaker overnight at 30°C at a rotation speed of 60 rev min^−1^. After incubation, 45 ml of the cultures was centrifuged at 5000*g* (Beckman Coulter Allegra X22R) for 20 min and the supernatant filtered through 0.45 *μ*m sterile syringe filters (Sarstedt) to remove any bacteria and collect bacteriophage.

### 2.6. Bacteriophage Sensitivity Test

An 800 *μ*l aliquot of each bacterial isolate was inoculated on marine agar with a micropipette and spread over the entire plate in order to form a bacterial lawn and allowed to dry for about 30 min. A 20 *μ*l aliquot from each of the bacteriophage-enriched filtrates was spot-inoculated on each bacterial lawn. Plaques radiating from the phage-enriched filtrate were considered as a positive phage infection of the bacterial lawn. Control plates without bacterial lawns were prepared and inoculated with phage-enriched filtrate drops from both sources (as above) in order to check for contamination of the water samples.

## 3. Results

### 3.1. Bacterial Identification

Seventy-one bacterial isolates were tested for antibiotic resistance (30 from ReefHQ Turtle Hospital, 20 from Fitzroy Island Turtle Rehabilitation Centre, 13 from Magnetic Island coastal water, and eight from Orpheus Island coastal water). Eleven of the bacterial strains that showed multidrug resistance to a minimum of three antibiotics were identified to at least the genus level. The predominant isolates were* Vibrio* spp. (73% of selected bacteria). Three (27%) of the selected bacteria were* Pseudomonas* spp. (Table 2).* Vibrio harveyi* and* V. parahaemolyticus* were two potential pathogens identified to species level (Table 2).

### 3.2. Antibiotic Susceptibility

**Figure 1:**
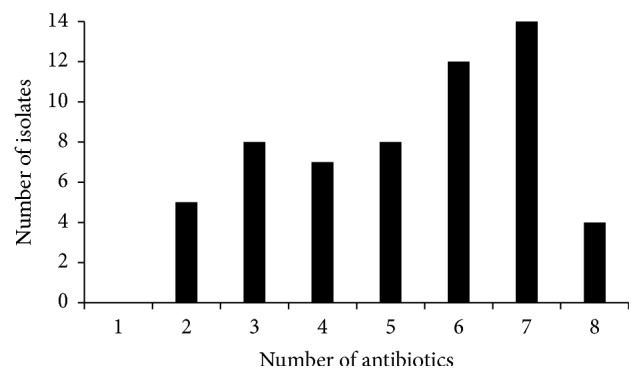
Frequency of resistant bacterial isolates to the eight antibiotics tested.

All isolates were resistant to at least two antibiotics, with 24% being resistant to seven of the eight antibiotics tested (Figure 1). The antibiotics that encountered the highest prevalence of resistance were ampicillin (range 40 to 69.2%), enrofloxacin (range 16.6–77%), amoxicillin plus clavulanic acid (range 15 to 62.5%), and ceftiofur (range 10–53.8%). An unexpected high prevalence of resistant bacteria was found in the coastal waters compared with the turtle's eye: 77% in water from Magnetic Island and 62.5% in that from Orpheus to enrofloxacin and 53.8% and 62.5% resistance in each coastal water, respectively, to amoxicillin and clavulanic acid (Table 2). Resistance prevalence to erythromycin was greater in the eye bacteria: 53.3% from ReefHQ and 50% from Fitzroy, as against 38.5% in coastal water from Magnetic Island and 12.5% from Orpheus. Resistance prevalence to doxycycline and thus all tetracycline antibiotics was moderate in isolates from ReefHQ (30%) and Magnetic Island (23%) and low for isolates from Fitzroy (5%) and Orpheus (0%). Low rates of antibiotic resistance were detected to chloramphenicol (range 0–10%) and trimethoprim/sulfadiazine (0–16.5%). In fact, there was no resistance to chloramphenicol observed in isolates from Orpheus and Magnetic Islands shore water and no resistance to trimethoprim/sulfadiazine observed in isolates from Magnetic Island shore water.

### 3.3. Bacteriophage Spot Tests

Seventy-two percent (72%) of phages lysed at least their targeted bacteria. All the bacteria, including* V. harveyi* and* V. parahaemolyticus, *were lysed by at least one of the phage filtrates (Table 3). The ReefHQ phage filtrate lysed only one of the bacteria, which was also lysed by 3 Thailand filtrates. Two of the filtrates from Thailand (#1 and #6) appeared to have lysed up to 4 bacterial isolates (Table 3).

## 4. Discussion

In this study, samples were taken from green turtle eyes in order to test for the presence of antibiotic resistant bacteria and their susceptibility to phage, an alternative treatment method. Throughout the existing literature, bacterial samples from sea turtles have been predominantly Gram-negative strains taken from the nasopharyngeal and cloacal area [10–12]. Gram-negative aerobic bacteria were also isolated from the tanks of two sea turtle rehabilitation centres in the North Queensland region and from the near-shore waters of islands in the region. The ReefHQ sea turtle hospital in Townsville treats debilitated sea turtles with five antibiotics, namely, amoxicillin trihydrate, enrofloxacin, trimethoprim/sulfadiazine, chloramphenicol, and oxytetracycline. The Fitzroy Island sea turtle rehabilitation centre in Cairns utilises enrofloxacin and oxytetracycline for the hospitalised sea turtles.

It is expected that environments where antibiotic use is high will select for a high level of antibiotic resistance in isolated bacteria [13, 14]. Therefore, the higher number of multidrug resistant bacteria from the rehabilitation centres was not surprising. However, the resistance to some individual antibiotics was higher in coastal water bacterial isolates. One would expect isolates from ReefHQ and Fitzroy Island Rehabilitation Centres to have the greatest prevalence of resistance to the broad-spectrum antibiotics enrofloxacin (a fluoroquinolone) and oxytetracycline (a tetracycline antibiotic). The moderate prevalence of resistance to enrofloxacin at both ReefHQ and Fitzroy Island Rehabilitation Centres was therefore expected, as was the lack of resistance to doxycycline in the coastal waters. Furthermore, the higher prevalence of the beta-lactam drugs and potentiated sulphonamides (trimethoprim/sulfadiazine) in the ReefHQ isolates compared to those from Fitzroy Island is also expected. What was unexpected was the high prevalence in coastal waters of bacteria resistant to the fluoroquinolones and broad-spectrum beta-lactam drugs.

Environmental bacterial resistance to quinolones (nalidixic acid), the parent drug of the fluoroquinolones such as enrofloxacin, has been found to be more common in reports from the Mediterranean Sea [15, 16] than in the Southern Eastern United States [17] or Australia [18]. Thus, we would expect to find a low prevalence of resistance to the fluoroquinolones, especially in coastal waters. This was not the case, since, as Table 2 shows, both sea water samples had higher numbers of resistant bacteria (e.g., 77% of bacteria isolated from coastal water off Magnetic Island) than the rehabilitation centres, and the results show a higher prevalence of resistance than previous antibiotic surveillance reports from Australia [19]. Although testing for resistance genes was not performed, bacteria in the small sample size of the waters would have been in contact and it is known that environmental bacteria rapidly develop resistance to these antibiotics [20], mainly because they can be reservoirs for the plasmid-mediated QnrS-like quinolone resistance determinants [21]. Globally a high level of tetracycline-resistance has been encountered in bacteria found in surface waters. Doxycycline, our test antibiotic, shows* in vitro* complete cross-resistance with oxytetracycline, as it has the same mode of action. In our study, bacterial strains appeared to be relatively susceptible to doxycycline (between 0 and 30%), despite higher resistance prevalence being reported in other studies from aquaculture farms [21]. Higher prevalence of tetracycline resistant genes may be expected in aquaculture farm waters, where tetracycline is used more intensively [22]. That aside, the highest prevalence of resistance in isolates from ReefHQ was expected considering the usage of this antibiotic while bacteria from Orpheus Island coastal water, which was expected to have the least antibiotic resistance, were completely susceptible to doxycycline. High resistance rates to penicillin and macrolide antibiotics have been encountered in a number of other studies. This is often due to intrinsic resistance of most Gram-negative bacteria [23]. Our study showed a lower prevalence of resistance to erythromycin (see Table 2) compared to a similar study from South Carolina (United States), which found that in rescued sea turtles the most frequent resistances of Gram-negative isolates were to erythromycin (95.2%) and penicillin (95.2%) [17]. As we had a predominantly Gram-negative bacterial population, we chose to test antibiotic susceptibility to beta-lactam drugs with a broader spectrum of activity such as ampicillin, ceftiofur, and amoxiclav. High prevalence of resistance to ampicillin and amoxiclav was found in studies on antibiotic resistance in bacteria from aquaculture sources in Australia (54.8% of isolates resistant to ampicillin) [18], from wild loggerhead turtles in the Mediterranean Sea (77.8% of isolates resistant to amoxicillin) [15], and from nesting green turtle in the Oman Sea (~65% of isolates resistant to ampicillin) [1]. Our results are similar to the Australian study and lower than the other studies. The number of beta-lactam drug resistant bacteria was higher from the coastal waters. Low resistance rates to ceftiofur have been reported from aquaculture farms in Australia [18]. This is in contrast with the high resistance detected in bacterial strains from the two North Queensland coastal islands isolated in this study. Ceftiofur is a third-generation cephalosporin beta-lactam drug that has a preferred spectrum of activity against Gram-negative bacteria. Therefore, bacteria developing extrinsic resistance to beta-lactam drugs could be expected to show similar patterns of resistance to this antibiotic and to the other beta-lactam drugs. Akinbowale et al. [18] found low levels of resistance to chloramphenicol (6.7%), attributing this result to the fact that in Australia it has been removed from use in livestock since 1982, whereas Foti et al. [15] found nearly 40% resistance to this drug in the Mediterranean Sea. All of these authors express concern at the spread of multidrug resistant bacteria in the environment, in aquaculture facilities, and in marine animal rehabilitation centres. Results in this experiment support this general concern regarding the fluoroquinolone, tetracycline, and beta-lactam classes of antibiotics. However, it was gratifying to note that, despite the use of chloramphenicol and potentiated sulphonamides at ReefHQ, the prevalence of resistance to them was low. The isolates from the rehabilitation centres showed a low level of resistance (10%), which may be attributed to occasional use of chloramphenicol eye ointment to treat eye disease. Bacterial isolates from both coastal waters showed no resistance to chloramphenicol. Although, as expected, bacterial resistance to potentiated sulphonamides was highest in isolates from ReefHQ, the percentage of resistance was surprisingly low considering that this antibiotic is often used for empirical therapy, particularly of respiratory tract infections. In addition, some of the highly resistant organisms isolated in this study were primary and opportunistic pathogens causing various diseases in marine organisms* (V. harveyi)* and gastrointestinal illness in humans* (V. parahaemolyticus)* [24, 25]. Moreover, a recent study discouraging sea turtle meat consumption in Mexico found several human pathogens, including* V. parahaemolyticus*, from wild sea turtles to be highly drug resistant [26]. Ninety-four percent of the* V. parahaemolyticus* isolates proved to be resistant to at least one commonly prescribed antibiotic and mainly to ampicillin, confirming the presence in coastal environments of multidrug resistant pathogens transmissible from turtles to humans. The potential for zoonotic disease transmission in captive sea turtle environments is discussed by Arena et al. (2014) [27] in a study on a green turtle farm in the Cayman Islands, where tourists closely interact with captive animals. Their findings included the isolation of several potential human pathogens such as* V. alginolyticus*,* Pseudomonas aeruginosa*, and* Escherichia coli*. The possible role of sea turtles as carriers of human pathogens is also discussed in a recent study which found two strains of* V. parahaemolyticus* on stranded sea turtles along the coast of Italy [28]. Little literature exists on bacteria isolated from sea turtle eyes [7, 8, 29]. Gram-negatives were more commonly isolated, reflecting our results (Table 2). Bacteria in the eyes of sea turtles were isolated from lesions caused by keratoconjunctivitis, ulcerative blepharitis, ulcerative keratitis, heterophilic scleritis, and salt gland adenitis. Several potential pathogens were present, including aeromonads and pseudomonads. These potential pathogens were found to be multidrug resistant in other locations [1, 15, 17, 18, 30]. In this experiment, eyes were considered of particular importance as their contact with the environment may make them good indicators of water quality. Our findings add to the existing concern about antibiotic resistant bacteria in wild and captive sea turtle environments and emphasise the need for antibiotic alternatives to optimise therapies in marine reptile rehabilitation centres, which in turn will reduce the quantity of active antibiotics found in effluents and of antibiotic resistant bacteria on the epithelia of turtles that are returned to the ocean.

Phages have been successfully implemented as antibiotic alternatives in humans [31], marine fish, invertebrates [32], poultry, and cattle [33]. Our results also show the efficacy of phage-enriched filtrates in clearing multidrug resistant bacterial strains. We found most of the tested bacteria were lysed by phages originating from aquaculture effluents (Table 3). This was predictable, as the aquaculture effluents were bacterially richer than the turtle tank water and the greater the bacterial density in an environment, the greater the phage diversity [34]. Three of the phage filtrates appeared to be highly specific, only lysing strain 4, a* Pseudomonas* species, and strain 11, a* Vibrio* species. There is a possibility that some of the phages are broad-spectrum, lysing closely related bacteria; for example, #6 shows lysis of closely related* Vibrio* species only and #4 targets only* Pseudomonas* species. As the phage filtrates were not purified, more species of phages may have been present, including temperate phages that did not lyse the bacteria or virulent phages with longer latency periods and therefore did not have the time to produce plaques during the 24 h incubation period. This may explain why some phage filtrates cleared bacteria other than the ones they were enriched with. The apparent broad host range of some phage-enriched filtrates—for example, phage filtrate #9 lysed both a* Vibrio* and* Pseudomonas* species (Table 3)—may be explained in the same way. Further isolation and purification of the filtrates are needed in order to understand the phages' host range and select lytic life-cycle phages only. This is possible as previous studies have successfully isolated and purified virulent phages that have lysed multidrug resistant strains of* V. harveyi* and* V. parahaemolyticus *originating from aquaculture facilities in Australia and have been able to successfully treat laboratory mice experimentally infected with* V. parahaemolyticus* [31, 35]. Phage therapy has also been proven highly successful when applied directly onto an infected body part (e.g., skin, ear, and oral cavity), which may particularly suit the topical treatment of eye infections in sea turtles [36]. Furthermore, in the case of enteric infections, thanks to their high host specificity, phages could be selected to clear only the targeted bacteria without affecting the turtles' beneficial microflora [37]. Moreover, phages would be safe to discharge in turtle rehabilitation centre water effluents, as once the density of their host bacteria is reduced, they too will decrease in number [38]. In the existing literature, phages have been recommended as antibiotic alternatives for a variety of animals [31–33].

As mentioned above, in this study phage filtrates were not purified and plaque formation was considered to be the result of lysis by phages as the plaques showed amplification and not dilution (which would have happened in the presence of other antimicrobial agents such as bacteriocins [39, 40]) when repeatedly cultured. Nevertheless, in order to add confidence to our findings, additional analyses will have to be performed and phage filtrates will have to be purified and characterized. Given our results, we suggest further investigations with the aim of using phage therapy on sea turtles in North Queensland rehabilitation centres, not only to control bacterial diseases but also to reduce the load of antibiotic resistant bacteria.

## 5. Conclusion

The prevalence of antibiotic resistance in the bacterial strains investigated in the North Queensland sea turtle rehabilitation centres and coastal island waters appears to be at alarmingly high levels for most antibiotics tested. Further investigations are needed in order to understand how bacteria in rehabilitation centres have developed resistance to antibiotics not used in those centres. Multidrug resistant bacteria may already be present in the coastal water taken up by the sea turtle hospitals, suggesting environmental pollution by antibiotics. As the enriched phage filtrates produced plaques in all the bacterial strains isolated, this study provides promising results on the efficacy of virulent phages in infecting bacteria present in sea turtle environments. The practice of assessing bacterial susceptibility to antibiotics prior to administration in order to provide the most effective treatment is crucial, and we emphasise the need for clinical trials and ongoing research into bacteriophage therapy as antibiotic alternatives for sea turtles.

## Figures and Tables

**Table 1:** Epidemiological cut-offs (mg/l) for the eight antibiotics tested and the initial working concentrations (mg/l) of the eight antibiotics used in the MIC tests.

	Antibiotic agents^1^							
	Am/c	Amp.	Cef.	Chl.	Dox.	Enr.	Ery.	Tm/s
Epidemiological cut-offs (mg/l)	>8/4	>4	>2	>16	>8	>0.25	>2	>0.5/9.5
Initial conc. (mg/l)	128/64	64	16.2	64	35.5	16	33.2	32/608

^1^Am/c: amoxicillin/clavulanic acid; Amp.: ampicillin; Cef.: ceftiofur; Chl.: chloramphenicol; Dox.: doxycycline; Enr.: enrofloxacin; Ery.: erythromycin; Tm/s: trimethoprim/sulfadiazine.

**(a):** 

Number of isolates (*n* = 30) at a MIC (mg/l) (ReefHQ)																										
Antibiotic agent	≤0.002	0.004	0.008	0.015	0.031	0.062	0.125	0.25		0.5		1	2		4		8		16		32	64	128	256	>256	% res
Amoxi./clav.					6			1		3		2	3		2		3	**|**	**5**			**2**	**3**			33.3
Ampicillin				8						3			2		2	**|**	**2**		**2**		**3**	**8**				50
Ceftiofur		8		1	1	2	1	1		3		2	6	**|**	**5**											16.6
Chloramphenicol				6			1			1		8	5		4		2			**|**	**1**	**2**				10
Doxycycline			2							3		2	1		1		7	**|**	**7**		**2**					30
Enrofloxacin		7	1	3	5	2	5	2	**|**	**3**		**1**							**1**							16.6
Erythromycin			6			1	1	2				1	3	**|**	**10**						**6**					53.3
Trim./sulf.			8	1	1	2	4	4		5	**|**	**1**	**2**		**1**				**1**							16.6

**(b):** 

Number of isolates (*n* = 20) at a MIC (mg/l) (Fitzroy)																										
Antibiotic agent	≤0.002	0.004	0.008	0.015	0.031	0.062	0.125	0.25		0.5		1	2		4		8		16		32	64	128	256	>256	% res
Amoxi./clav.					1					6		3	2		4		1	**|**	**1**			**1**	**1**			15
Ampicillin				1				1		2		3	4		1	**|**	**2**		**1**		**1**	**4**				40
Ceftiofur		2	1	3	1	1	2	2		3		2	1	**|**	**1**				**1**							10
Chloramphenicol				1				2		2		9	3		1					**|**	**2**					10
Doxycycline			1				1			2		8	4		3			**|**	**1**							5
Enrofloxacin		1			2	3	4	6	**|**	**3**		**1**														20
Erythromycin			1					1		4		4		**|**							**10**					50
Trim./sulf.			1		4	1	6	5		2	**|**	**1**														5

**(c):** 

Number of isolates (*n* = 13) at a MIC (mg/l) (Magnetic Island)																										
Antibiotic agent	≤0.002	0.004	0.008	0.015	0.031	0.062	0.125	0.25		0.5		1	2		4		8		16		32	64	128	256	>256	% res
Amoxi./clav.								1		1		1	1		1		1	**|**	**1**		**2**	**1**	**3**			53.8
Ampicillin										1		1	2			**|**	**1**		**1**		**2**	**5**				69.2
Ceftiofur						1	3	1		1				**|**	**1**		**3**		**3**							53.8
Chloramphenicol										1		2	1		1		5		3	**|**						0
Doxycycline										1		2	1		1		5	**|**	**3**							23
Enrofloxacin							2	1	**|**	**2**		**3**			**2**		**3**									77
Erythromycin					1		2			1		4		**|**							**5**					38.5
Trim./sulf.				4	5	3	1				**|**															0

**(d):** 

Number of isolates (*n* = 8) at a MIC (mg/l) (Orpheus)																										
Antibiotic agent	≤0.002	0.004	0.008	0.015	0.031	0.062	0.125	0.25		0.5		1	2		4		8		16		32	64	128	256	>256	% res
Amoxi./clav.					3													**|**	**1**		**1**	**2**	**1**			62.5
Ampicillin				3												**|**			**1**			**4**				62.5
Ceftiofur		3								1				**|**	**3**				**1**							50
Chloramphenicol				2								1	4		1					**|**						0
Doxycycline			2					1					1		3		1	**|**								0
Enrofloxacin		2						1	**|**	**2**					**1**		**2**									62.5
Erythromycin			3									1	1	**|**							**1**					12.5
Trim./sulf.			3					3		1	**|**	**1**														12.5

^1^ReefHQ Turtle Hospital, Fitzroy Island Rehabilitation Centre, Magnetic Island, and Orpheus Island. ^2^Vertical bars (|) are the epidemiological cut-offs (numbers in bold after | are counted as antibiotic resistant isolates). Last column is the percentage of resistant bacteria (% res) for each antibiotic.

**Table 3:** Bacterial susceptibility to 11 bacteriophage filtrates.

Bacterial strain	Source^1^	Bacteriophage filtrates^2^										
		#1	#2	#3	#4	#5	#6	#7	#8	#9	#10	#11
(1)* V. harveyi*	TE						L1		L1			
(2) ***Pseudomonas* sp.**	TE				L1							
(3)* V. orientalis*	TE			L1								
(4) ***Pseudomonas* sp.**	TT		L1		L1						L2	
(5)* V. harveyi*	DT	L1				L1	L1		L1			
(6) *V. cyclitrophicus*	DT	L1					L1					
(7)* V. harveyi*	MI	L1					L1	L1				
(8)* V. parahaemolyticus*	MI	L1					L1		L1			
(9) ***Pseudomonas* sp.**	TE				L1					L1		
(10)* V. nereis*	TE			L1						L1		
(11)*** Vibrio* sp.**	FI	L1										L1

^1^TE: ReefHQ green turtle eye, TT: ReefHQ green turtle tank, DT: ReefHQ display tank, MI: Magnetic Island water, and FI: Fitzroy Island turtle tank. ^2^Phage filtrates numbers (#1–11) correspond to their isolating strains ((1)–(11)) (L1: lysed by phage filtrate sourced from Thailand aquaculture waste waters and L2: lysed by phage filtrate sourced from ReefHQ green turtle tank water).
